# Impact of Physical Growth and Development on Paediatric Lower-Limb Prosthetic Provision: Prosthetist Perspectives and Clinical Casefile Analysis From Cambodia

**DOI:** 10.1177/27536351251384354

**Published:** 2025-10-27

**Authors:** Claudia Ghidini, Caitlin E. Edgar, Carson Harte, Sisary Kheng, Anthony M. J. Bull

**Affiliations:** 1 The Centre for Paediatric Blast Injury Studies, Department of Bioengineering, Imperial College London, UK; 2 Exceed Worldwide and The Department of Prosthetics and Orthotics, Phnom Penh, Cambodia

**Keywords:** paediatric, prosthetic care, growth, socket fit, prosthetic length discrepancy

## Abstract

**Introduction:**

Growth affects prosthetic provision in children, leading to socket fit issues and prosthetic length discrepancy. Despite increasing numbers of paediatric amputations, no studies have systematically analysed clinical casefiles or interviewed prosthetists to identify growth-related challenges and mitigation strategies, particularly in low-resource environments where polypropylene (PP) technology is used. This study addresses this gap by interviewing prosthetists and analysing clinical casefiles.

**Methods:**

This study combined qualitative interviews with Cambodian prosthetists and analysis of 62 clinical casefiles. Casefile analysis documented growth-related issues, adjustment methods, and time between interventions. Thematic analysis was applied to interviews.

**Results:**

Five themes were identified, highlighting that: sockets and their use can account for growth through careful oversizing and using liners/socks; handcrafted adjustments that rely on thermoplastic technology can also accommodate for growth; lack of adjustment increases waste and clinical attendance; poor socket fit causes pain and residual limb problems; and growth issues result in universal problems of socket fit issues and prosthetic length discrepancy.

**Discussion:**

This study is the first to interview paediatric prosthetists to assess growth-related challenges, identify mitigation strategies, and combine these qualitative findings with hard clinical evidence. PP prosthetic systems offer cost-effectiveness and increased adjustability compared to modular components and carbon fibre sockets. However, this adjustability is still limited, leading to waste of resources and increased clinical time. Finally, longer-than-recommended replacement timelines are concerning, and more research is necessary to understand these longer delays. Addressing these limitations is crucial, particularly in low-resource environments, to improve accessibility and prevent secondary impairments.

## Introduction

Function and prosthetic provision after lower-limb amputation in children is significantly affected by growth, resulting in socket fit issues and prosthetic length discrepancy (PLD).^1^ These issues may cause discomfort, lead to the development of residual limb conditions^1^, other secondary impairments such as lower back pain^2,3^ and potentially misuse. The lack of adjustability in current paediatric prostheses not only increases clinical burden on children, their families, and clinicians, but also leads to a significant waste of resources and negatively impacts residual limb health.^1^ Affordable, adjustable sockets and pylons for children are in development,^4-8^ however these are not yet commercially available.

Despite the significant increase in the numbers of paediatric amputations and the lack of research in the field,^9,10^ no studies to date have interviewed paediatric prosthetists to identify the perceived challenges caused by growth during paediatric prosthetic provision and the solutions they implement,^11^ nor have any detailed analyses of clinical casefiles been conducted to address this knowledge gap. Due to their work, prosthetists have expertise in available technologies, the needs of their patients and the most recurrent challenges with prosthetic devices.^12^ Indeed, they have been extensively interviewed to develop questionnaires for adult prosthetic users or to clinically drive future research questions.^13,14^ A recent study has identified the major challenges in paediatric prosthetic provision identifying growth as the main driver for clinical attendance but have not dived into the specific solutions implemented by prosthetists to address issues arising from growth.^1^ It has only been anecdotally reported how prosthetists address these problems, and with a focus on prosthetic modular systems and other technologies, mainly available in high-resource environments (eg, flexible inner sockets).^15-18^ No research to date has investigated the methods used to adjust for growth using the prosthetic technology most commonly available in low-resource environments and conflict zones: polypropylene (PP) technology developed by the International Committee of the Red Cross (ICRC).^19^ Additionally, recommended frequency of adjustments and replacements due to growth are reported in literature (every 3-6 and 12-18 months respectively)^15-18,20-22^ but have never been estimated using actual clinical data. It is crucial to accurately estimate the actual frequency of attendance, and assess whether the reported timelines realistically reflect how often children and their families can visit the centre, particularly in low-resource environments.

The lack of research led by clinical data and prosthetists’ specialised knowledge hinders provision for children and slows down development of prosthetic devices that meet the needs of the key stakeholders: the child and the prosthetist. Additionally, during sudden onset disasters such as earthquakes or in conflict, many children can suffer from traumatic amputations and prosthetists may find themselves treating a cohort they are not familiar with and who pose unique challenges for provision.^23-25^ Creating comprehensive guidelines on the most prevalent problems and possible solutions for growth is critical, especially to improve disaster preparedness by including this knowledge in current rehabilitation guidelines.

The aim of the study is to address the knowledge gap in prosthetist strategies to support childhood growth by interviewing paediatric prosthetists and analysing reported problems and solutions from clinical casefiles of children with lower-limb absence.

The objectives of the study are to:

- identify perceived growth-related challenges in paediatric provision through qualitative interviews and understand their impact on child wellbeing and residual limb health,
- identify the solutions implemented to address these challenges by complementing qualitative interviews with casefiles analysis, and
- quantify the waste of resources and increased clinical access associated with lack of adjustability of paediatric prostheses.

## Methods

This study was approved by the National Research Ethics Committee of Cambodia (NECHR_24) and the local institutional ethics committee in the UK (ICREC_6609720).

This study complemented qualitative interviews with prosthetists in Cambodia with an analysis of clinical casefiles of children with lower limb absence in Cambodia. These methods are presented separately below.

### Casefile Analysis

Sixty-two casefiles of children with a major lower limb absence (transtibial, knee disarticulation, and transfemoral level) due to any aetiology, collected by the Department of Prosthetics and Orthotics (DPO) and Exceed Worldwide, were analysed as part of this study. Casefiles contained information on patient’s demographics and reasons for clinical visits (eg, growth, durability, and residual limb issues) from 2005 until 2023. Individual participant consent and legal guardian consent were waived, because the study utilised anonymised clinical data.

Previously these casefiles were used to analyse the main drivers for clinical attendance, including growth, prosthetic durability, and residual limb issues.^1^ In this study, only the clinical notes related to growth, specifically those mentioning growth, prosthesis tightness, or prosthesis shortness, were analysed in detail. The 2 main problems caused by growth, socket fit tightness, and prosthetic length discrepancy were addressed by either full prosthetic replacement or a prosthetic adjustment. Prosthetic adjustment was defined as a modification of the current prosthesis (either socket or height) to accommodate for growth.^1^ Replacements were defined as complete prosthetic replacements necessitated by the child having fully outgrown their current device.^1^ The type of adjustment methods employed to address each issue were recorded and percentage per type was estimated. A combination of different adjustments could be employed to address a problem, so the number of adjustment solutions may be higher than the number of problems. The time between successive adjustments and replacements was also quantified as a measure of necessary clinical access for growth.

Finally, the number of times each type of growth-related problem resulted in a replacement or adjustment was quantified to assess if different issues resulted in different amounts of replacement. To analyse the relationships between the 2 categorical variables, the Chi-Square test for independence was run with alpha set at .05.^26^ If the assumption for Chi-Square on lowest expected cell frequency was violated, the Fisher’s Exact Probability Test was run.^26^ For post-hoc test, the Bonferroni adjustment to the alpha was applied to control for type error 1 (alpha/number of sub-comparisons).^26^

### Qualitative Interviews

Prosthetists were recruited from 4 prosthetic centres across 4 separate Cambodian provinces through purposive sampling. Interviews were held online over a video-call or in-person at the prosthetic centre. The interviews followed the protocol laid out by Edgar et al^11^ and spanned many domains, including growth. When necessary, a translator was available.

Prospective participants were provided with a full verbal explanation of the study and a participant information sheet, and were given sufficient time to ask questions. If they agreed to take part, they were asked to sign a consent form, including permission to audio-record the interviews for subsequent transcription and interpretation.

Audio-recordings were transcribed using the Microsoft Word transcriber (Version 2409) followed by manual correction by the interviewer. The interviewer familiarised themselves with the interview and open-coded them following the thematic analysis method by Braun and Clarke.^27^ Similar codes were merged. However, the number of times a code was repeated was kept ensuring data representation.

Finally, merged codes were filtered in MATLAB (R2022a, MathWorks) to identify only those relevant to the macro-area of growth. The following words were used to filter the code database: ‘growth’; ‘prosthetic length discrepancy’; PLD; ‘fit’; ‘volume’; ‘measurements’; ‘stump’; ‘residu’; ‘distal end’; ‘adjust’; ‘skin mark’; ‘circumfer’; ‘longitude’; ‘skin’; ‘length of prosthesis’; ‘grow’; ‘heating’; ‘socket’; ‘liner’; ‘trimline’; ‘cuff’; ‘ICS’; ‘casting’; ‘grinding’; ‘pe-lite’; ‘suspension’; ‘EVA’; ‘foam.’ Subsequently, the source data was checked to ensure no irrelevant code was accidentally included or omitted.

The final codes were then merged into themes and reviewed by 2 researchers independently. Codes repetition that supported the theme were summed whereas repetition of contrasting codes was subtracted to ensure an accurate representation of the prosthetists’ opinion (eg, 5 repetition of ‘Growth causes socket tightness problems’ and 2 repetition of ‘Socket is not a problem’ would result in a theme with importance 3).

## Results

Six prosthetists were recruited as part of this study from 4 different centres. Three centres were affiliated with the Department of Prosthetics and Orthotics (DPO) supported by Exceed Worldwide. The ICRC supported 1 centre. The prosthetists have worked an average 8.6 ± 6.6 years with children with limb absence: 5 of them were senior (⩾5 years of experience) and 1 was junior (<5 years). Half of the interviews were conducted remotely, and the other half on site at the prosthetic centres. Interviews lasted 83 (SD = 12) minutes.

The demographic information of the 62 casefile is provided in table 1. For children with acquired amputation, average age at amputation was 10.1 ± 4.5 years.^1^

**Table 1.** Casefiles Demographics (Reproduced in Part From Ghidini et al^1^).

Variable	% (n), N_TOT_ = 62
Year of birth	
1991-1995	8.1 (5)
1996-2000	17.7 (11)
2001-2005	32.3 (20)
2006-2010	22.6 (14)
2011-2015	16.1 (10)
2016-2020	3.2 (2)
Sex	
Female	27.4 (17)
Male	72.6 (45)
Type of prosthesis supplied^a^	
Transfemoral	35.5 (22)
Knee disarticulation	9.7 (6)
Transtibial	54.8 (34)

a Due to the complexity of congenital limb absence, the level of prosthetic components supplied is used as a proxy for limb absence level to account for children who may not have had elective amputations as part of their limb difference treatment.

The prosthetists provided care to all levels of paediatric lower limb absence, although partial foot and hip disarticulation amputations were reported to be rare. All 4 prosthetic centres provided PP technology free of charge. The PP system was sourced from a factory owned by the Persons with Disability Foundation of Ministry of Social Affairs, Veterans and Youth Rehabilitation, Cambodia. In some of the centres, modular paediatric components were also available free of charge as they were donated by a charity. Any other modular component was available through personal finance.

All prosthetists reported providing in general self-suspended patellar tendon bearing sockets for transtibial cases, self-suspended push fit sockets for knee disarticulation cases and ischial ramus containment sockets with Silesian belt for transfemoral cases. In general, children were provided with Ethylene-Vinyl Acetate (EVA) foam as a liner, also known as PE-lite liner. Silicone liners were also available in some of the centres. Children were provided with solid ankle cushion heel (SACH) feet and prosthetic knee joints (PP monocentric knee joint, polycentric modular knee joint) if they presented with above or knee disarticulation amputation. The following 5 themes were identified and are summarised below: socket design and manufacture can account for growth, growth adjustability is handcrafted and relies on polypropylene technology, lack of growth adjustability increases waste and clinical attendance, poor socket fit causes pain and residual limb problems and assessing goodness of socket fit is extremely challenging, socket fit issues and prosthetic length discrepancy are universal problems, yet growth is individualised.

### Challenge 1: Socket design and manufacture can account for growth

Prosthetists highlighted that during the rectification stage of socket manufacturing, they take preventative measures to increase the lifespan of the socket and accommodate for growth of the residual limb.

The importance of finding the right balance between socket fit, control, and ease of donning and doffing the prosthesis was highlighted multiple times. Socket fit should be slightly loose to prolong the lifespan of the prosthesis; however, if the fit is too loose, it may hinder the child’s ability to effectively control the device. During the rectification process, the socket is made bigger and then cotton socks are used to ensure a secure fit in the initial months. A socket with a snug fit will be difficult to wear for children and may not last as long, so 5 out of 6 prosthetists advised against it. Additionally, considering distal end build-up during socket rectification is crucial to prevent discomfort or pain from the residual limb touching the distal end of the socket as the child grows. This is also useful for cases of bone overgrowth.

Oh of course of course, normally we need to consider about growth when we make the socket. That’s why from my experience when I make the socket, I normally make it a little bit loose. . . .. Normally we use 12-millimetre EVA to use at the distal end of the positive mould for children. . . So, if the length of the stump grows or there is bone spur or something like that, we can take that distal layer/pad out. So, we can spare maybe 5 or 8 millimetres [at the distal end].

This one [making socket loose] is for both: easy donning and doffing and also maybe the socket lasts a bit longer. Because the children grow up very fast, that’s why we change the socks only and then we will not make the new device for them. We can put 2-3 layers, something like that and then we can remove socks. Also we can pad EVA foam on proximal pad, mostly they’re concerned at the suspension area. And then for distal end, we just keep some space for growing - 2-3cm something like that.

Another important consideration is the socket trimlines, which should not be cut too short during manufacturing to prevent the self-suspension method from quickly becoming ineffective as the residual limb outgrows the socket.

Yeah. So, we have to get the trimline really accurate, so we do not get it too short otherwise when the person grows, then it is getting shorter and shorter. The patient is growing, right? But the socket cannot grow.

However, it is important to find the right balance as trimlines that are too long will disturb the child during play and other activities.

Usually, we follow the standard procedure like supracondylar suprapatellar suspension, but sometimes it a bit high for them and will stop them from doing their activities. But if we do not make like that the socket will not suspend then that’s why it’s a problem for the child.

### Challenge 2: Growth adjustability is handcrafted and relies on polypropylene technology

Regardless of the measures taken during socket fabrication, prosthetists mentioned how children do come back to the clinic with growth-related problems in their prosthesis. During the interviews, prosthetists listed some of the methods they employ to address socket tightness and prosthetic length discrepancy.

The 2 most common methods reported to address socket tightness were swapping cotton socks and heating out the PP socket. Indeed, as children grow, cotton socks are swapped to maintain a secure socket fit, which was initially made slightly loose, as explained above. Heating out the PP socket was also reported as an effective method to cope with growth as the PP material can be easily expanded in specific areas. Prosthetists also highlighted the residual limb does not grow uniformly; therefore, only specific areas of the socket require modification.

Yes, usually for adjustment we do heating in the socket. Because normally, when it is tight it is not the whole area that is tight. . . . So, most of the time that the client comes and then the socket feels tight so, we do the heating, especially at the bony areas like head of fibula and the condylar area and most of the time the area where we do the heating a lot is the distal end.

Other methods to address socket fit issues are adding/removing EVA padding which can be used to increase self-suspension or relieve pressure on some areas, especially distal end. Some of the EVA liner material can also be ground off if necessary.

So then when the distal end is touching the liner we can take out the additional EVA pad.

To address prosthetic length discrepancy, prosthetists have fewer strategies available. The main method consists of adding a block of PP at the ankle connection: the foot is un-welded and unscrewed, the extra block is added and then the foot is welded and screwed back.

So for one thing, when we deliver the whole device after like they used it for like 3 months or six months and then they feel it is short we can adjust something but only at the ankle part, add a piece of block at the ankle part, but only one centimetre, 1 centimetre approximately. If it’s more than that, we will need to change prosthesis.

Alternatively, they can pad extra EVA foam under the SACH foot. However, prosthetists highlighted the latter method is not ideal as it is custom in Cambodia to walk barefoot in the house and sometimes outside as well, therefore the padding would wear out quickly.

So, when the height is different, I just pad the EVA underneath in one side.

So, in here, in our culture we’re not wearing the shoes inside the house. And they will go out sometime barefoot so the height of intact and prosthetic side must be the same.

During the interviews, it also became evident prosthetists in low-resource environments prefer the PP system for children as it allows easy adjustments for socket tightness (heating out) and prosthetic length discrepancy (adding PP block at ankle level). Prosthetists believe it is the best alternative for children when considering adjustability provided, price point, and the high rate of prosthetic replacements due to growth.

No, in my opinion in here if we use like carbon fibre or anything, it’s difficult to adjust, to make small adjustment. Like if you want to heat, in terms of heat, polypropylene is easier for that [than carbon fibre]. This is like another way of saving material, something like that. Because you know child will grow up more, so if we provide something like carbon fibre or anything, it’s expensive and our way to like change or adjust [the socket] is less.

The analysis of the adjustment methods employed by prosthetists and reported in the clinical notes of the casefiles matched their reported techniques as seen in table 2.

**Table 2.** Adjustment Techniques for Polypropylene System Reported in the Notes of Clinical Casefiles.

Problem	Type of adjustment	Number	%
Socket tight	Heating socket	16	36.4
	Grinding material off socket/liner	12	27.3
	Swapping socks	12	27.3
	Removing/Adding EVA pad	2	4.5
	Cutting trimlines	1	2.3
	Unspecified	1	2.3
Prosthetic length discrepancy	Adding PP Block at ankle level	27	75.0
	Unclear^a^	3	8.3
	Adding rubber under prosthetic foot	2	5.6
	Adding EVA pad inside shoe	1	2.8
	Changing prosthetic foot^a^	1	2.8
	Unspecified	2	5.6

a Entry that refers to the modular component available at the centre.

### Challenge 3: Lack of growth adjustability increases waste and clinical attendance

Even with the reported techniques to address growth, the adjustments are sometimes insufficient, and component waste can occur.

If the socket is just a bit tight maybe we can try grinding some material out/away. If it is short, too short, we have to make a new one, but if it is just a bit short, maybe we can try to accommodate with some padding under the shoe or adjust on the prosthesis. . .. But if the problem is big like now, the patient could not put the leg inside the socket, then we make a new one.

**Figure 1.**
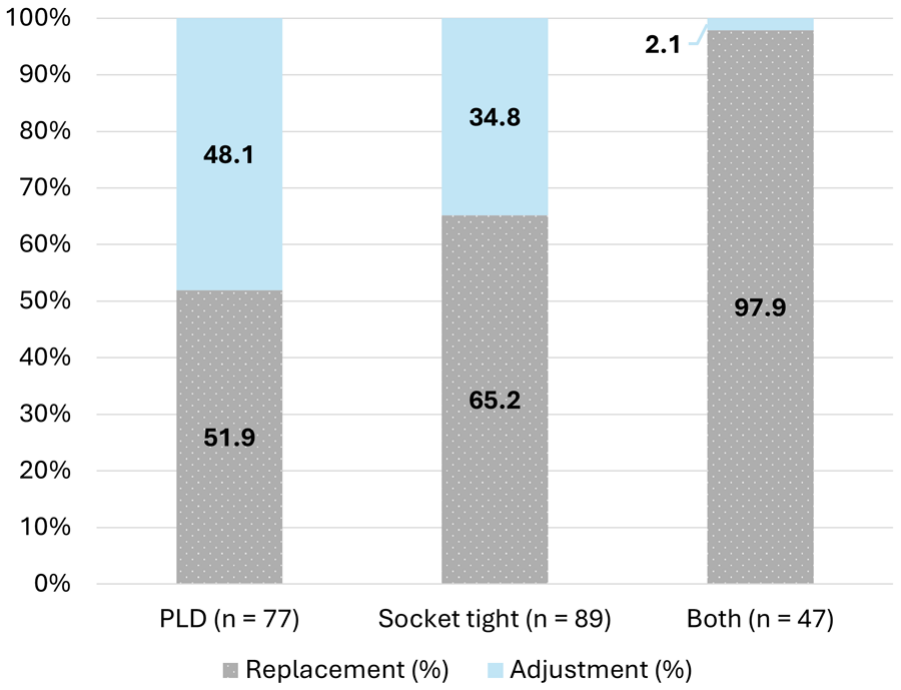
Proportion of replacements and adjustments for prosthetic length discrepancy (PLD), Socket tightness, both problems together. (Solid: Prosthetic Adjustment; Dotted: Prosthetic Replacement).

The proportion of replacements and adjustments based on problem encountered are reported in Figure 1. When both problems (tightness and PLD) presented themselves together, prosthetists had to completely replace the prosthesis in 97.9% of the visits. The Chi-Square test for independence found a significant association between problem encountered (socket tightness, PLD, and both problems) and solution implemented (replacement and adjustment), χ^2^(2, n = 213) = 28.521, *P* < .001, phi = .366. Post hoc analysis with *P*-value set at .008 (.05/number of comparisons) revealed there were less adjustments than expected when both socket tightness and PLD occurred (*P* < .000001), and more adjustments than expected for only the group PLD (*P* = .0002).

Prosthetists highlighted the need for increased clinical access due to growth and the fact that sometimes children come back before the scheduled appointments presenting with the growth issues.

Yes, sometimes they come [before the scheduled appointment] for pain, okay? Or device is short. And also, because the device becomes small because they become bigger and bigger. They come to change the device.

Casefiles showed that the adjustment in socket or prosthetic length occurred on average every 8.3 (±6.6) months, whereas full prosthetic replacements due to growth occurred on average every 16.4 (±9.9) months. This was supported by the prosthetist’s opinions.

Yeah, mostly adjustments six months. OK, because three months is not a big difference. Six months to one year: during this time, we need to do some adjustment. After one year, we need to change to a new one.

### Challenge 4: Poor socket fit causes pain and residual limb problems and assessing goodness of socket fit is extremely challenging

Prosthetists described the impact of the socket fit challenge throughout growth. They reported poor socket fit causes residual limb conditions in children such as skin marks, redness on bony prominences or weight bearing areas (ischium and patellar tendon), skin discoloration and pain in the residual limb. Indeed, visually checking for these conditions is the number 1 method to assess if children have socket fit problems as often direct communication is challenging. For instance, children struggle to say where they have pain, they just say that they have pain, therefore it is key to establish effective communication with them and their caregivers. It was also highlighted that pain and skin issues are localised rather than uniform across the residual limb.

Skin mark maybe. Skin mark and socket fit. . . . And also, we look on the weight bearing area. Like if it puts the pressure on the wrong place.

Sometimes socket fit is also the problem because they could not tell us that it is loose, is it tight, is it comfortable? Whatever. So, it is mainly dependent on what we have to check. . . . They just say ‘chou chou’ which means pain and we had to find out by ourselves what is causing the pain. . . . So, it’s a bit like we need really strong communication skills with the kid, otherwise it’s not success.

Another method to assess poor fit is checking the number of socks in use as sometimes sock misuse causes socket fit challenges. Also, prosthetists check previous measurements collected and lifespan of current prosthesis to determine if socket fit may already be a problem.

We always consider about how long has the child used the device. And also, we will check the stump. Sometimes they complain about the tightness of the socket, but they use for example three or four layers of the sock. So, we try to ask them to use only one sock to see.

Checking gait deviation to assess goodness of fit and alignment is sometimes complicated with young children (less than 5 years of age) as they run around and do not really walk on a straight line when asked to, therefore prosthetists highlighted the need for a more standardised method to assess socket fit.

You cannot just tell them to walk straight. They just walk following their feelings. That’s why we cannot check easily.

### Challenge 5: Socket fit issues and prosthetic length discrepancy are universal problems, yet growth is individualised

Prosthetists confirmed that the 2 fundamental issues caused by growth are socket fit tightness and prosthetic length discrepancy and all children experience these issues.

Most of the time the problem that we got when they grow up is the length of the prosthesis. Also, the socket fitting: as I already said most of the time it is tight. But especially the length of the prosthesis is shorter than the intact limb and we need to adjust.

However, prosthetists highlighted multiple times that all children are different. They do not all grow at the same rate and require individualised care. Although growth-related problems, solutions and residual limb health impacts are relevant for the entire cohort, it was repeatedly mentioned each child will experience these challenges at different ages and may respond differently. As a result, it is important to treat each patient as a unique individual and avoid generalising care.

From the small to like around 10 years old, they did not grow very fast, right. But between 10 to 14 year and then in that time they have a growth spurt. Yeah. Yeah, yeah. Below 10 they grow, but not as fast as like between 10 to 14 years old.

Yeah, we do not have that [a standardise time to replace prosthesis] because the child grows differently, each individual child.

## Discussion

This is the first research study to interview paediatric prosthetists to identify the challenges associated with growth during paediatric prosthetic provision.^11^ The methodology used qualitative interview outcomes to then investigate the arising themes quantitatively using clinical casefiles.

Prosthetists highlighted how accounting for growth in this cohort throughout provision is of paramount importance. The initial step in accommodating growth involves creating a larger prosthetic socket with a deliberately loose fit, utilising thick cotton residual limb socks to ensure the socket remains secure and comfortable. This technique has been anecdotally reported in literature.^15,16^ However, for the first time it was found this loose fit not only accommodates for growth but also facilitates independent donning and doffing of the prosthesis by the child.^21^ Communicating the appropriate techniques for donning and doffing a snug-fitting socket can present unique challenges when working with young children, thus implementing techniques in socket manufacture can aid this process and provide confidence to the child especially during early rehabilitation. Supporting children’s independence and confidence is crucial for their development: enabling them to manage the wearing and removal of their prosthesis without constant parental assistance empowers them, such as when they need to use the toilet at night. The importance of finding the right balance between loose fit, prosthetic control, and ease of donning/doffing was mentioned. While a socket that is too loose may hinder the control and functionality of the prosthesis,^28,29^ an excessively tight fit could restrict comfort and ease of independent use as well as reduce lifespan due to growth.

Additionally, when possible, children in low resource environments receive self-suspended sockets which rely on suspension from bony prominences,^19^ and this is the common provision in the centres in this study. This approach minimises the number of required components, thereby reducing the complexity of the prosthesis and mitigating issues related to the durability^1^ and cost of individual components. However, with self-suspended sockets, it is critical to avoid shortening the socket trimlines excessively, as doing so may compromise the self-suspension method as the child outgrows the socket, leading to a full socket replacement. This is particularly important to consider during manufacturing for prosthetists who may have limited access to other suspension methods such as pin lock systems, sleeves, or lanyards. However, other prosthetists mentioned trimlines that are too long may restrict children’s ability to play and participate in daily activities. It is critical to find the right balance to prolong prosthesis lifespan whilst enabling the child to comfortably use the prosthesis. Finally, prosthetists highlighted the importance of considering build-up at the distal end of the socket to accommodate longitudinal growth of the residual limb, but also for a unique residual limb condition children suffer from: bone overgrowth. Bone overgrowth is defined as ‘abnormal development of appositional new bone at the distal end of the residual limb’ and can cause severe pain and prevent prosthetic use.^30^ Adding removable EVA pads at the bottom of the socket creates room in these cases and can prolong prosthetic use before surgical intervention.^23,31,32^

Regardless of the preventive measures taken, children will inevitably come back to the prosthetic centre requiring adjustments and replacements of their prosthesis to accommodate for growth. For the first time, all the methods employed by prosthetists to adjust the PP prosthesis were identified and quantified using the casefiles. The methods listed in the casefiles matched those mentioned during the qualitative interviews. The most common methods to address socket tightness and prosthetic length discrepancy are swapping cotton socks and heating out the PP socket to modify the shape and adding a PP block at the ankle level to correct length discrepancy. Some of the methods employed are hand-crafted and successful implementation is based on experience and skills.

Although some adjustments are possible for a prosthesis that is too short and/or too tight, the limited growth-adjustability of current prosthetic solutions results in a significant waste of resources. In this study, prosthetic length discrepancy and socket tightness together contribute to a higher rate of replacements (97.9%). Compared to an adjustment (1 visit), a full prosthesis replacement requires at least 3 clinical visits (casting, fitting, and delivery). Therefore, the burden on children, their families, and clinicians significantly increases, as highlighted by prosthetists and as previously found.^1^ Additionally, this study found that the PP system allows for increased adjustability when the prosthetic length discrepancy problem arises, a benefit that has not been previously identified. Indeed, prosthetists mentioned the PP system is better for children when considering the rate of prosthetic replacements in this cohort, its cost-effectiveness, and the flexibility it provides in addressing both socket tightness and prosthetic length discrepancy, which is not provided by the carbon fibre sockets and metal pylons. Indeed, only 1 adjustment to address prosthetic length discrepancy was reported in the casefiles when using the modular system. Furthermore, local factories in Cambodia can melt and recycle 60% to 70% of the polypropylene used in prosthetic centres,^1^ helping to mitigate the environmental impact of the frequent replacements in this cohort. This does not apply to carbon fibre, which cannot be recycled. Despite this, carbon fibre is lighter, which is beneficial for children.^15,30^ Additionally, adjustable metal pylons do exist but are still not widely available and quite expensive. Therefore, the prosthetic industry should still focus on developing technologies that combine both advantages to better meet the ever-changing needs of this cohort.

Furthermore, the time passed between adjustments and replacements, estimated using the casefile clinical notes, is longer than what is reported as appropriate in literature (3-6 and 12-18 months respectively)^15-18,20-22^ It is important to investigate the reasons behind these longer times. Using an poorly fitting prosthesis has been reported to lead to secondary impairments in the adult cohort.^2,3^ It can be speculated that these impairments may be more significant in this cohort as the developing musculoskeletal system is still plastic and responds more readily and dramatically to altered loads (such as seen in cerebral palsy^33^). It is essential to not only investigate the impact that these altered loadings may have on the musculoskeletal system, but also to develop prostheses that can accommodate for growth and can be adjusted outside of clinical settings. Such innovation would guarantee improved prosthetic fit and potentially mitigate against onset of these secondary impairments, especially for children living in settings where access to care may be limited. Preliminary research on developing affordable adjustable prostheses for children exists but it is in the early stages^4-8^ and not tested to the international ISO standard for prostheses.^34^

Prosthetists have also noted that socket fit issues can lead to problems in children’s residual limbs, such as wounds and skin discoloration, which is supported by previous research.^1^ However, assessing socket fit can be challenging due to communication barriers and the difficulty in getting children to walk in a straight line for gait deviation evaluation. Building trust with both children and their families from the start is crucial for ensuring effective communication, which can in turn help identify potential fitting issues. However, improving current methods to assess socket fit in this cohort warrants attention to prevent the residual limb problems related with poor socket fit. A recent study on including children in research and prosthetic design identified the Socket Comfort Score questionnaire as a potentially appropriate outcome measure for this cohort.^11,35^

The knowledge gained in this paper is valuable for disaster preparedness and as guidelines for junior prosthetists who may have little experience working with children. Yet, each child is unique and deserves individual consideration to provide optimal care as the timing of problem occurrence, the best communication methods, and their preferences for optimal care likely differ. This is equally applicable for children with different amputation level as anatomy of the residual limb, prosthetic design, and prosthetic components differ significantly. This is further supported by previous research which identified prosthetic length discrepancy as a universal problem in the cohort, whereas children with transtibial and knee disarticulation amputation are more likely to experience socket tightness compared to transfemoral cases.^1^

This study has some limitations. Although supported by the casefile analysis, qualitative interviews are subjective, increasing biases; this was mitigated by the rigorous methodology followed. Additionally, only 6 prosthetists have been recruited. However, this sample size was chosen as previous research found that 73% of thematic codes are identified with 6 interviews.^11,36^ Furthermore, prosthetists working in private centres in Cambodia have not been interviewed, potentially overlooking some strategies to address growth issues when using technologies other than the PP system. The geographical focus on Cambodia may restrict the generalisability of the findings to other contexts. However, challenges associated with PP prosthetic technology and solutions implemented are relevant to similar settings globally, as this technology is also available and used in other regions. Lastly, due to insufficient data for each age group and sex, it was not possible to investigate the difference in the timing of growth spurts.

In conclusion, this is the first time prosthetists have shared their perspectives on the challenges related to growth during paediatric prosthetic provision and how to address them. The polypropylene system is considered suitable for children due to its cost-effectiveness and level of adjustability provided. However, the prosthetic industry should focus on developing technologies that combine these advantages with the benefits of modular systems and carbon fibre sockets, which are lighter weight. Lastly, the qualitative interviews were supported by hard clinical evidence, which highlighted the significant waste of resources associated with growth and delayed access to care. This study provides valuable insights into the unique intricacies of paediatric prosthetic provision, which can be used by clinicians and prosthetic providers alike to better serve the needs of this vulnerable group of patients.
